# Accuracy of valganciclovir dosing following kidney transplantation and its impact on clinical outcomes

**DOI:** 10.3389/fneph.2026.1781576

**Published:** 2026-06-26

**Authors:** Jessica Forsyth, David Randall, Raj Thuraisingham

**Affiliations:** Department of Renal Medicine & Transplantation, Royal London Hospital, Barts Health National Health Service Trust, London, United Kingdom

**Keywords:** cytomegalovirus, drug monitoring, kidney transplant, prophylaxis, valganciclovir

## Abstract

Valganciclovir is widely used as prophylaxis against cytomegalovirus (CMV) infection following solid organ transplantation. However, valganciclovir requires dose adjustment based on kidney function. This retrospective observational study at a London kidney transplant center explored the accuracy of valganciclovir dosing in the first 3 months following transplantation and its impact on clinical outcomes. Data for adult kidney transplant recipients (KTRs) receiving valgancivloir prophylaxis between 2017 to 2019 was analyzed. The primary outcome was the difference between the prescribed and optimum valganciclovir dose (calculated by creatinine clearance) on days 10–92 after surgery. Secondary outcomes included associations between valganciclovir dosing with CMV DNAemia, cytopenias and rejection. For the 90 KTRs included, the valganciclovir dose was optimum for 51%, underdosed for 31% and overdosed for 18% of the study period. Significant associations were shown for underdosing and CMV DNAemia (B = 2.88, SE = 1.14, p=0.01, 95% CI [1.90,166.1]) as well as overdosing and neutropenia (B = 2.29, SE 0.99, p=0.02, 95% CI [1.42,68.71]). This study highlights the difficulty in optimally dosing valganciclovir in KTRs post-operatively and importantly shows significant associations between suboptimal dosing and clinical outcomes.

## Introduction

Cytomegalovirus (CMV) is a common opportunistic pathogen following solid organ transplantation (SOT), imparting significant risk of post-operative morbidity and mortality (1, 2). Most clinically significant CMV infection (csCMVi) occur in the first months following transplantation (3, 4). Valganciclovir is widely used as prophylaxis for csCMVi following high-risk SOTs (5, 6). In addition, valganciclovir can treat breakthrough csCMVi at higher doses.

Optimal dosing is crucial; overdosing risks iatrogenic bone marrow suppression and cytopenias, while underdosing risks csCMVi and development of valganciclovir resistance (5–8). Valganciclovir is renally cleared and therefore dosing is renally adjusted. This presents a challenge in kidney transplant recipients (KTRs) where kidney function is particularly dynamic post-operatively.

This study assessed the proportion of days within the first 3 months post-operatively in which KTRs received optimally dosed valganciclovir, as determined by creatinine clearance (CrCl). It also explored associations between suboptimal valganciclovir dosage and clinical outcomes. The hypothesis was that over- and underdosing with valganciclovir may increase the risk of cytopenias and breakthrough CMV DNAemia (respectively).

## Materials and methods

### Study design

This was a single-center retrospective observational study conducted out of the Renal Transplant unit at the Royal London Hospital in London, United Kingdom. Electronic patient records were analyzed from an outpatient transplant clinic service and screened for KTRs aged 17 and older between April 2017 and June 2019. KTRs were included where criteria for valganciclovir prophylaxis were met, as per local guidelines (see below). Simultaneous pancreas-kidney transplants and hyperacute graft failure were excluded.

### Outcomes

The primary outcome was the proportion of days post-operatively where patients were optimally dosed, underdosed or overdosed on valganciclovir. Secondary outcomes included the incidence of CMV DNAemia, leucopenia, neutropenia and graft rejection.

### Definitions & local guidelines

Local transplant guidelines stratified recipients according to immunological risk. Recipients were deemed high immunological risk and received anti-thymocyte globulin (ATG) induction where: 1) donor-specific antibodies (DSAs) were over 1400 mean fluorescence intensities (MFIs), 2) a previous transplant was rejected with historical or current DSAs or 3) cold ischemic time was >24 hours. All other recipients received basiliximab induction.

Local guidelines recommended prophylaxis with valganciclovir post-operatively according to induction agent and CMV status. Prophylaxis was administered for 3 months in CMV D+/R- KTRs receiving basilixmab, and for six months in CMV D+ and/or R+ KTRs receiving ATG.

Local guidelines recommended dosing valganciclovir according to CrCl. CrCl was used in-line with guidelines by the British National Formulary, specifically to increase dosing accuracy given the relatively narrow therapeutic window of valganciclovir (9). In this study, creatinine was standardized by an isotope dilution mass spectrometry assay by Roche and CrCl calculated by the Cockcroft-Gault equation: ((140-age(years)) x weight(kg) x constant)/creatinine (micromol/L). Prophylactic dosing was as follows: 900mg once daily for CrCl >60ml/min, 450mg once daily for CrCl 40-59ml/min, 450mg alternate days for CrCl 25-39ml/min, 450mg twice weekly for CrCl 10-24ml/min and 450mg once or twice weekly for CrCl <10 or on dialysis.

CMV DNAemia is defined as the detection of CMV DNA in blood or plasma. In this study, plasma CMV was measured using a PCR amplification assay by Roche. Local guidelines recommended treatment for CMV DNAemia where plasma viral load >3000 IU/ml. Treatment was continued for a minimum of 2 weeks or until two consecutive negative viral loads were achieved. Treatment dosing was as follows: 900mg twice daily, 450mg twice daily, 450mg twice daily, 450mg once daily, and 450mg thrice weekly respectively for the CrCl categories defined above.

Transplant recipients attended clinic twice weekly post-operatively for the first 6–8 weeks, then weekly until 12 weeks. CMV viral loads were measured weekly and kidney function twice weekly until 12 weeks. Transplant clinicians were responsible for confirming valganciclovir dose at each clinic by selecting an electronic ‘tick box’ or re-prescribing the dose.

Leucopenia was defined as white cell count (WCC)<3x10^9^/L and neutropenia <2x10^9^/L. Rejection was defined according to the contemporaneous Banff criteria at time of biopsy. Valganciclovir serum levels were not assessed in-line with national and international guidelines for routine clinical practice.

### Data collection

Demographic data was extracted for (1) age at transplant, (2) sex, (3) transplant type, (4) induction agent, (5) donor CMV status and (7) recipient CMV status. For outcomes, data was extracted for (1) creatinine, (2) weight, (3) CMV viral load, (4) valganciclovir dose, (5) WCC, (6) neutrophils, (7) histological rejection and (8) dialysis sessions. Data was analyzed from days 10 to 92 following transplant; this period was selected due to the high incidence of acute CMV DNAemia and dynamic changes in kidney function post-operatively. The first 10 days were excluded due to paucity of outpatient data in the immediate post-operative period.

CrCl was calculated from creatinine and actual weight for every data entry and then intrapolated across the study period. For other data categories, data was inferred from the most recent reading where there was a data gap. For inclusion, patients required >/=5 measured CMV viral loads and >/=10 confirmed valganciclovir doses. Outpatient data had to cover >/=60 days of the study period cumulatively. Days of viraemia and dialysis were included in analysis.

The optimum valganciclovir dose was calculated for each day in the study period (according to CrCl and CMV viral load) and compared to the prescribed dose, categorizing each day as either optimally dosed, underdosed or overdosed. The percentage of days for each patient in each dosing category was used in analyzing secondary outcomes. In addition, the number of actual dose changes over the study period per patient was compared to the number of ideal dose changes.

### Statistics

Data was analyzed using SPSS v29. Associations between transplant type and valganciclovir dosing were analyzed using one-way ANOVA and Bonferroni-corrected pairwise comparison where relevant. Associations between valganciclovir dosing and CrCl were analyzed by simple linear regression, and CMV status and induction agents with independent T-tests. Associations between adverse outcomes and adverse outcomes and transplant type were analyzed by Chi-squared tests. Associations between valganciclovir dosing and adverse outcomes were analyzed with binomial logistic regression. A p<.05 denotes statistical significance and 95% confidence intervals (CI) are reported.

## Results

### Patient demographics

99 patients met all criteria for valganciclovir prophylaxis. 9 were excluded due to insufficient data; 2 cases with insufficient CMV entries, 6 cases with <60 days of data and 1 case due to both the former and insufficient valganciclovir dose entries. For the 90 patients included in the study, there was a median of 18 valganciclovir dose entries (IQR 16-19) and a median of 19 CMV entries (IQR 17-21).

**Table 1** highlights patient characteristics. The indication for valganciclovir prophylaxis was use of ATG induction in CMV D+ and/or R+ in 62 patients (68.9%) and basiliximab in CMV D+/R- in 28 patients (31.%). The median age was 49 (IQR 38-57).

**Table 1:** Patient characteristics, transplant details and incidence of adverse outcomes.

Variable	Characteristic	N (=90)	%
*Age at transplant*	17 - 29	11	12.2
	30 - 44	25	27.8
	40-54	40	44.4
	60 - 74	14	15.6
*Sex*	Male	57	63.3
	Female	33	36.7
*Transplant type*	DBD	50	55.6
	DCD	27	30.0
	LD (U)	4	4.4
	LD (R)	9	10.0
*Induction agent*	ATG	62	68.9
	Basiliximab	28	31.1
*CMV status* *(donor/recipient)*	+/+	22	24.4
	+/-	37	41.1
	-/+	16	17.8
	Unknown/+	12	13.3
	Unknown/-	3	3.3
*Incidence of HD (from day 10 post-operatively)*	Patients requiring HD for any number of days	3	3.3
*Patients experiencing adverse outcomes*	Any adverse outcome	39	43.3
	CMV viraemia	12	13.3
	WCC <3.0	23	25.6
	Neutrophils <2.0	19	21.1
	Transplant rejection	3	3.3

ATG, anti-thymocyte globulin; DBD, donation after brain death; DCD, donation after cardiovascular death; LD, live donor; U, unrelated; R, related; HD, haemodialysis; CMV, cytomegalovirus; WCC, white cell count.

### Valganciclovir dosing

**Figure 1:**
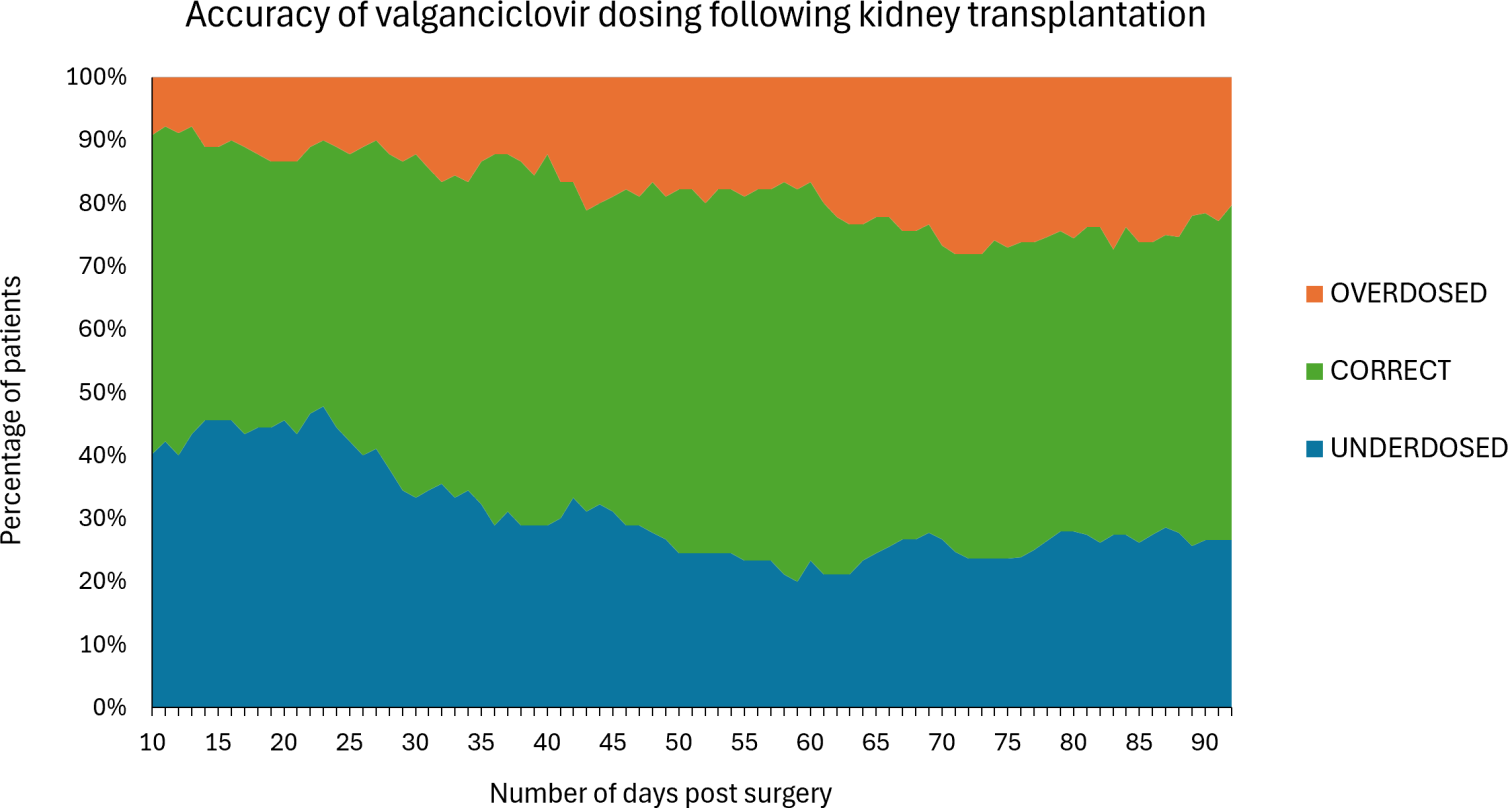
Percentage of patients underdosed, overdosed and optimally dosed on valganciclovir CMV prophylaxis for the first 10–92 days following kidney transplantation.

Patients on valganciclovir spent an average of 51% (95% CI [44%, 58%]) of days optimally dosed, 31% (95% CI [24%, 37%]) of days underdosed and 18% (95% CI [13%, 24%]) of days overdosed (N = 90) (**Figure 1**). Patients were more likely to be underdosed than overdosed immediately after surgery: at day 12, 40% were underdosed and 8% overdosed, compared to day 90, where 27% were underdosed and 21% overdosed.

There were significant associations between sub-optimal valganciclovir dosing, transplant type and renal function.

Transplant type significantly associated with valganciclovir dosage (Oneway ANOVA: F(2,92)=3.79, p=0.03). Donation after cardiovascular death (DCD) KTRs spent more time underdosed than live donor (LD) KTRs (Mean 38.6% vs 11.9% respectively); this was non-signficant on comparison with donation after brain death (DBD) KTRs (Mean 31.3%) (Bonferonni: DCD vs LD p=0.03, DCD vs DBD p=0.65, LD vs BDB p=0.73). There was no significant association for time overdosed (F(2,89)=1.58, p=0.21).

CrCl varied significantly by transplant type (Oneway ANOVA: F(1,90)=4.18, p=0.04). CrCl was significantly lower in DCDs compared to LDs (Mean 50.1 vs 66.5 respectively; Bonferonni p=0.04, 95% CI[-32.08,0.85]), but non-significantly for DCDs vs DBDs (Mean 55.2 for DBD, Bonferonni p = 0.78).

CrCl significantly associated with valganciclovir dosage, with lower average CrCl associating with time overdosed (Linear regression; F(1,89)=12.17, p=<0.01; B=-0.01, b=-0.35, t(89)=-3.49, p=<0.01, 95% CI[-0.08,-0.02]) and trending to significance for time underdosed (F(1,89)=3.42, p=0.06, 95% CI [-0.07, 0.00]).

There were no significant associations between valganciclovir dosing and induction agent, age or CMV status.

### Dose adjustments

The average number of actual dose adjustments per patient was 1.16 (95% CI [0.92,1.39]) whereas the average number of optimum dose adjustments was 4.10 (95% CI [3.37,4.83]). This suggests an average of 2.94 optimum dose adjustments did not occur; there was no significant difference between optimum dose increases and decreases that did not occur (Mean: 1.40 vs 1.54 respectively, t(89)=-1.37, p=0.17, 95% CI [-0.35, 0.07]).

There was significant association between average CrCl and optimum dose adjustments (Linear regression: F(1,89)=15.68, p< 0.01). Lower average CrCl was associated with more optimum dose adjustments (B=-0.07, beta=-0.39, t(89)=-3.96, p<0.01, 95% CI [-0.10,-0.04]). There was no significant association between optimum dose adjustments and transplant type (F(2,89)=2.77, p=0.07), induction agent (F(1,89)=2.48, p=0.11) or age (F(1,89)=0.83, p=0.37).

### Clinical outcomes

Just under half of all patients (n=39, 43.3%) experienced at least one adverse outcome in terms of CMV DNAemia (n=12, 13.3%), drop in WCC <3 (n=23, 25.6%), drop in neutrophils <2 (n=19, 21.1%) or transplant rejection (n=3, 3.3%).

On analysis of transplant demographics, there were no significant associations between type of transplant, induction agent, age or CMV status with incidence of any individual adverse outcome. Of interest, higher risk CMV +/- recipients had no significantly increased incidence of CMV viraemia than other CMV donor states (16.2% vs 11.3%; X2 (1,90) = 0.452, p=0.50).

**Table 2** shows significant associations between valganciclovir dosage and clinical outcomes. An association was found between valganciclovir dosage and CMV DNAemia (X2(2)=7.28, p=0.03), where time underdosed was positively associated with breakthrough CMV DNAemia (B = 2.88, SE = 1.14, p=0.01, 95% CI [1.90,166.10]). There was a significant association between valganciclovir dosage and neutropenia (X2(2)=5.60, p=0.05), where time overdosed positively associated with increased incidence of neutropenia (B = 2.29, SE 0.99, p=0.02, 95% CI [1.42,68.71]). Leucopenia had no significant association with valganciclovir dosage (X2(2)=1.91, p=0.38). Rejection was too infrequent to assess significant associations (N = 3).

**Table 2:** Effect of valganciclovir dosage on clinical outcomes.

Variable	Time spent underdosed				Time spent overdosed			
	B	SE	p	95% CI	B	SE	p	95% CI
*Any outcome*	0.24	0.74	0.74	0.30 – 5.41	**3.17**	**1.21**	**<0.01**	**2.22- 256.02**
*CMV viraemia*	**2.88**	**1.14**	**0.01**	**1.90 – 166.11**	1.27	1.43	0.37	0.22 – 58.17
*WCC drop < 3*	0.12	0.72	0.87	0.26 – 7.50	1.20	0.86	0.15	0.59 – 20.87
*Neutrophil drop < 2*	1.11	0.95	0.25	0.47 – 19.56	**2.29**	**0.99**	**0.02**	**1.42 – 68.71**

B, unstandardized coefficient; SE, standard error; CI, confidence interval; p is significant <0.05 (denoted in bold).

There were significant associations between adverse outcomes. CMV DNAemia was associated with increased incidence of leucopenia (X2(1)=4.25, p=0.04, N = 88) and neutropenia (X2(1)=6.94, p<0.01, N = 90).

## Discussion

This retrospective observational study found that valganciclovir was optimally dosed for only 51% of the time during the first months following kidney transplantation. Our study showed that suboptimal valganciclovir dosage significantly associated with adverse outcomes: time spent underdosed associating with breakthrough CMV DNAemia, and time spent overdosed associating with neutropenia.

Patients with lower renal function required more optimal dose changes and spent more study time incorrectly dosed, identifying this as a vulnerable patient group. DCDs had lower average CrCl and were more likely to be incorrectly dosed. The increased prevalence of underdosing directly after transplant likely reflects the dynamic changes in renal functions during this time, also highlighting this as a crucial time for drug monitoring. The significant difference between the number of optimum and ideal dose changes highlights missed dose adjustments by clinicians as a key area of concern.

These findings both supplement and challenge those of a study published by the Swiss Transplant Group in 2024. In a double-center retrospective observational study from 2010-2020, this group assessed valganciclovir dosing in KTRs post-operatively and assessed association with breakthrough CMV DNAemia and cytopenias (10). They also showed KTRs are optimally dosed on valganciclovir for only half the time in the first few months post-operatively (44.6% of patient’s dose week-entries) (10). However, they did not find a significant association between underdosing and breakthrough CMV DNAemia (underdosing 15% vs optimally/overdosed 23.7%, p=0.44) (10). Of interest, their population had significantly more live donors than our study (38% vs 18% respectively). This is relevant given our findings that live donors are relatively less likely to be underdosed (likely because they achieve stable CrCl faster and have a higher average CrCl than deceased donor transplant recipients) and may therefore be at less risk of breakthrough CMV DNAemia. In addition, whilst they did not demonstrate an association with overdosing and neutropenia, they did demonstrate an association with lymphopenia (OR 5.27, 95% CI 1.71-16.22, p=<0.01) (10). Put together, these studies suggest that suboptimal valganciclovir may be a common problem internationally with the potential to impact clinical outcomes.

Our finding that time underdosed on valganciclovir associated with breakthrough CMV DNAemia adds weight to the ongoing debate about optimal valganciclovir prophylaxis dosing. Several recent studies have suggested that lower doses of valganciclovir prophylaxis (ie 450mg daily for eGFR >60ml/min) may have clinical equipoise in reducing CMV DNAemia to standard prophylaxis doses (ie 900mg daily), with reduced adverse side effects from cytopenias (11–14). However, other studies, including ours, have found significant CMV DNAemia breakthrough (6). The evidence from recent systematic reviews is inconclusive and several international guidelines do not support lower prophylactic valganciclovir dosage (5, 6, 8). Clearly larger studies are needed; attention to how confounders such as kidney function, induction agent, comorbidity and even genetics should be addressed to enable a personalized approach in guiding adoption of lower dose valganciclovir prophylaxis, either where there is less vulnerability to CMV or increased vulnerability to cytopenias.

## Limitations

Whilst every effort was made to maximize available data, there is a relatively small patient number in this study. The significant associations do not capture temporal relationship between valganciclovir dosage and adverse outcome, preventing causal interrogation. Compliance and intolerance were not assessed. Furthermore, suboptimal doses were categorized binarily as underdosed or overdosed rather than a quantitative deviation from ideal dose, which may have enabled more accurate associative analysis.

## Implications for future practice

There has been interest in CMV prophylactic agents that do not require adjustment to renal function, such as the hepatically cleared letermovir and maribavir (15). However, the cost on patent of both these drugs is relatively prohibitive (e.g average daily cost of letermovir is USD $251-$494 vs valganciclovir USD $11-$24) (16). In addition, letermovir and maribavir inhibit cytochrome P450 which increases serum levels of calcineurin inhibitors, thereby still necessitating costly and timely drug level monitoring. There is variable international and national guidance on these agents (6); for example, in the United Kingdom, letermovir prophylaxis is not recommended in national guidelines due to paucity of evidence on cost-effectiveness (17). Therefore, accurate valganciclovir dosing is likely to remain a critical issue for the near future.

Another solution is a stronger emphasis on antiviral stewardship initiatives. In a study by Hensler et al. in 2018, a SOT implemented a pharmacist-driven automated electronic monitoring system and performed a comparative retrospective study to assess the impact on the accuracy of valganciclovir prophylaxis dosing (18). The study found pharmacists reduced the 1-year incidence of breakthrough CMV DNAemia whilst on valganciclovir prophylaxis from 61% to 34% (p=0.03) as well as reducing ganciclovir-resistant CMV cases (18). Increasing antiviral stewardship initiatives in SOT centers may reduce morbidity and mortality associated with CMV, as well as addressing the international risk of viral resistance (19, 20).

## Conclusion

In summary, this study highlights significant inaccuracies in valganciclovir dosing following kidney transplantation, with implications for adverse clinical outcomes. The results of our study support the need for initiatives to improve prescribing practices in acute transplant clinics, including dedicated transplant pharmacists.

## Data Availability

The raw data supporting the conclusions of this article will be made available by the authors, without undue reservation.
